# Supplementary material for: A Comparative Analysis on the Biochemical Composition and Nutrition Evaluation of Crayfish (Procambarus clarkii) Cultivated in Saline-Alkali and Fresh Water
Source: Foods. 2025 Jun 5;14(11):1997. doi: 10.3390/foods14111997 (PMC12155198; doi:10.3390/foods14111997)
Supplement: Supplementary file 1 [file foods-14-01997-s001.zip › Table S1.pdf]

Table S1. The salinity, alkalinity, and pH of saline-alkali conditions for juvenile crayfish feeding.

	Salinity (‰)	Alkalinity (mmol/L)	pH
PC-SW	1.60	11.22	7.59
PC-FW	0.18	2.67	7.43
