# Supplementary material for: A Comparative Analysis on the Biochemical Composition and Nutrition Evaluation of Crayfish (Procambarus clarkii) Cultivated in Saline-Alkali and Fresh Water
Source: Foods. 2025 Jun 5;14(11):1997. doi: 10.3390/foods14111997 (PMC12155198; doi:10.3390/foods14111997)
Supplement: Supplementary file 1 [file foods-14-01997-s001.zip › Table S2. Method validation results for trace elements in crayfish.pdf]

Table S2. Method validation results for trace elements in crayfish.

	Standard curve	R^2	LOD (mg/L)	Recovery (%)	RSD (n=3,%)
Cu	y = 7294.3x + 213.46	0.9993	0.0100	80.0-105.0	< 10.0
Fe	y = 6976.8x + 237.25	0.9986	0.0100		
Zn	y = 6396.9x - 313.66	0.9992	0.0500		
Se	y = 2000000x - 1019.4	0.9991	0.0004		
K	y = 355.1x - 139.69	0.9991	3.0000		
Na	y = 350.73x - 140.95	0.9994	3.0000		
Ca	y = 525.01x - 143.26	0.9980	2.0000		
Mg	y = 517.92x - 288.16	0.9993	2.0000		
Mn	y = 5459.9x - 279.03	0.9966	0.0500		
